# Detection of Impaired IgG Antibody Formation Facilitates the Decision on Early Immunoglobulin Replacement in Hypogammaglobulinemic Patients

**DOI:** 10.3389/fimmu.2015.00032

**Published:** 2015-02-02

**Authors:** Hermann M. Wolf, Vojtech Thon, Jiri Litzman, Martha M. Eibl

**Affiliations:** ^1^Immunology Outpatient Clinic, Vienna, Austria; ^2^Department of Clinical Immunology and Allergology, Faculty of Medicine, Masaryk University, Brno, Czech Republic; ^3^Department of Clinical Immunology and Allergology, St. Anne’s University Hospital, Brno, Czech Republic

**Keywords:** hypogammaglobulinemia, IgG antibody deficiency, CVID, immunoglobulin treatment, IVIG, primary vaccination

## Abstract

Hypogammaglobulinemia (serum IgG lower than 2 SD below the age-matched mean) and clinical symptoms such as increased susceptibility to infection, autoimmune manifestations, granulomatous disease, and unexplained polyclonal lymphoproliferation are considered to be diagnostic hallmarks in patients with common variable immunodeficiency (CVID), the most frequent clinically severe primary immunodeficiency syndrome. In the present study, we investigated patients with hypogammaglobulinemia and no clinical or immunological signs of defective cell-mediated immunity and differentiated two groups on the basis of their IgG antibody formation capacity against a variety of different antigens (bacterial toxins, polysaccharide antigens, viral antigens). Patients with hypogammaglobulinemia and intact antibody production (HIAP) displayed no or only mild susceptibility to infections, while CVID patients showed marked susceptibility to bacterial infections that normalized following initiation of IVIG or subcutaneous immunoglobulin replacement therapy. There was a substantial overlap in IgG serum levels between the asymptomatic HIAP group and the CVID patients examined before immunoglobulin treatment. HIAP patients showed normal levels of switched B-memory cells (CD19^+^CD27^+^IgD^−^), while both decreased and normal levels of switched B-memory cells could be found in CVID patients. IgG antibody response to a primary antigen, tick-borne encephalitis virus (TBEV), was defective in CVID patients, thus confirming their substantial defect in IgG antibody production. Defective IgG antibody production against multiple antigens could also be demonstrated in an adult patient with recurrent infections but normal IgG levels. To facilitate early treatment before recurrent infections may lead to organ damage, the antibody formation capacity should be examined in hypogammaglobulinemic patients and the decision to treat should be based on the finding of impaired IgG antibody production.

## Introduction

A considerable percentage of patients seen in clinical practice, e.g., by ENT specialists for recurrent infections (1, 2) and/or referred for immunological evaluation have hypogammaglobulinemia, usually defined as a decrease in serum IgG lower than 2 SD below the age-matched mean, with a variable decrease in IgA and/or IgM serum levels. Common variable immunodeficiency (CVID) is the most frequent clinically severe primary immunodeficiency (PID) syndrome and the most common indication for lifelong immunoglobulin replacement therapy due to predominant antibody deficiency. CVID is believed to comprise a heterogeneous group of patients that have defective antibody formation in common while other known PID syndromes should have been excluded and substantial defects in cell-mediated immunity are lacking. In view of the known heterogeneity, diagnostic criteria for CVID are more and more under debate (3). A diagnosis of CVID has considerable clinical relevance, as it invariably results in long-term immunoglobulin replacement therapy, and the question of how defective antibody formation should be demonstrated is not uniformly clear. A serum IgG lower than 2 SD below the age-matched mean is considered to be one diagnostic hallmark in patients with CVID (4, 5). Although impaired antibody responses were included as a decisive diagnostic feature for CVID in PID classification reports very early on [e.g., as stated in Ref. (6):”The *sine qua non-for* the diagnosis of CVID is defective antibody formation.”], the most commonly used European Society for Immunodeficiencies/Pan American Group for Immunodeficiency (ESID/PAGID) definition of CVID (4) proposes hypogammaglobulinemia and demonstrable impairment in antibody responses as equivalent criteria, and it has even been reported that “positive vaccination responses are not contradictory to the diagnosis of CVID” (7).

In addition to hypogammaglobulinemia, the presence of clinical symptoms, such as increased susceptibility to infection, autoimmune manifestations, granulomatous disease, unexplained polyclonal lymphoproliferation, or an affected family member with antibody deficiency, is mandatory for the diagnosis of CVID in the 2014 registry diagnostic criteria for CVID proposed by experts in the field (5), given that all other forms of primary antibody deficiency and secondary forms of hypogammaglobulinemia can be excluded. Increased awareness for PID has been raised during the last decade with the ultimate goal of an earlier diagnosis and initiation of adequate therapy. This development is certainly desirable. Thus, patients with predominantly antibody deficiency such as X-linked agammaglobulinemia (XLA) who have a long history of clinical disease, in particular, recurrent infections of the lower respiratory tract, are well known to be prone to developing organ damage such as chronic lung disease, which determines their long-term prognosis (8). However, earlier presentation of patients with suspected PID also means that more and more patients with predominantly antibody deficiency lack a long history of clinical disease, making it necessary to initiate immunoglobulin replacement therapy based on laboratory findings rather than patient history. In view of this development, a more advanced laboratory definition of patients in need of intravenous immunoglobulin (IVIG) or subcutaneous immunoglobulin (SCIG) therapy is required than the one that is given, among others, in the currently used criteria for CVID diagnosis (4, 5).

In the present study, we performed an immunological investigation in patients with hypogammaglobulinemia and no clinical or immunological signs for defective cell-mediated immunity and differentiated patients with CVID requiring immunoglobulin replacement treatment from patients with hypogammaglobulinemia receiving no immunoglobulin therapy on the basis of their IgG antibody formation capacity against a variety of different antigens (bacterial toxins, polysaccharide antigens, viral antigens). IgG antibody response to a primary antigen, e.g., tick-borne encephalitis virus (TBEV) was examined in CVID patients already receiving IVIG therapy to reevaluate their IgG antibody production capacity. To further underline the importance of defining clinically relevant antibody deficiency by measuring antibody responses rather than serum-immunoglobulin levels, a patient was presented with a massive defect in IgG antibody production comparable to that seen in CVID despite normal IgG serum levels.

## Patients and Methods

### Patients with hypogammaglobulinemia and controls

Forty-nine patients with hypogammaglobulinemia defined as a serum IgG concentration below 500 mg/dl [median age (years) 37, interquartile range (IQR) 22–54, 26 men, 23 women], were included in a retrospective observational cohort study after the patients gave their informed consent that the anonymized data collected as part of the routine medical attendance the patients received could be included in a scientific publication. In these patients, no clinical or immunological indication of defective cell-mediated immunity (i.e., combined immunodeficiency) could be found. The patients had been referred for immunological investigation because of hypogammaglobulinemia and/or recurrent infections, recurrent fever of unknown origin, etc. (for a detailed description of the clinical symptoms in the individual patients, see Table 1) and were assigned to two groups depending on whether they were diagnosed as CVID according to the criteria established by the IUIS expert committee (6) and set on immunoglobulin replacement therapy or left untreated. Based on the results of the immunological workup such as determination of serum-immunoglobulin classes and IgG subclasses, IgG antibody titers to a variety of different antigens and/or antibody response after booster immunization it was found out that IgG antibody formation capacity distinguished these two groups of hypogammaglobulinemic patients, which correlated with the patients’ susceptibility to infections. The clinical characterization and serum-immunoglobulin levels of the 23 hypogammaglobulinemic patients [11 women and 12 men, median age at diagnosis (years) 41, IQR 19.5–61.5] with intact IgG antibody formation [hypogammaglobulinemia and intact antibody production (HIAP)] is given in Table 1 (A). This group of patients did not receive IVIG replacement. In 26 patients [14 men and 12 women, median age at diagnosis (years) 33, IQR 22.5–49.25, Table 2] CVID was diagnosed according to the criteria established by ESID (4, 5), and other PID disorders were excluded by sequence analysis (Illumina technology performed on a MiSeq bench-top next generation DNA sequencer) of PID genes listed in the 2011 IUIS classification (9). The CVID patients showed increased susceptibility to infections and [Table 1 (B)] and were treated with IVIG or SCIG, and blood samples for determination of serum-immunoglobulin levels and serum antibody concentrations were drawn before regular IVIG or SCIG replacement therapy was started. All results presented in this study were obtained as part of the routine medical attendance that the patients received; no extra venipuncture was performed on the basis of this study. Healthy adult blood donors served as controls.

**Table 1:** **(A) Clinical characteristics and serum-immunoglobulin levels in patients with hypogammaglobulinemia but intact IgG antibody production (HIAP); (B) Clinical characteristics and serum-immunoglobulin levels in CVID patients**.

Patient no.	Sex	Age at diagnosis (years)	Medical history leading to immunological evaluation	Serum immunoglobulins (mg/dl)		
				IgG	IgA	IgM
**(A)**						
1	M	6	Recurrent febrile episodes, hypogammaglobulinemia	360	40	95
2	M	9	Fever, diarrhea, abnormal liver function tests, hypogammaglobulinemia	291	18	26
3	M	22	Allergic rhinitis, hypogammaglobulinemia	393	64	120
4	M	25	Recurrent febrile episodes, hypogammaglobulinemia	459	115	127
5	F	37	Family history of antibody deficiency, hypogammaglobulinemia	431	162	201
6	M	34	Rec. Rhinitis, rec. gastritis, knee hurts, hypogammaglobulinemia	349	36	60
7	M	50	Nephrolithiasis, hypogammaglobulinemia	379	128	36
8	F	72	Hyperthyreosis, recurrent rhinitis	466	245	167
9	M	72	Chronic prostatitis, hypogammaglobulinemia, recurrent herpes labialis	427	530	71
10	F	89	Chronic fatigue, hypogammaglobulinemia	414	38	142
11	F	54	Chronic fatigue, hypogammaglobulinemia, recurrent sore throat, recurrent UTI	497	35	84
12	M	17	Celiac disease, herpes zoster, hypogammaglobulinemia	444	55	67
13	F	68	Chronic bronchitis, adrenal adenoma, hypogammaglobulinemia	430	139	171
14	M	8	Recurrent febrile episodes, hypogammaglobulinemia	466	112	112
15	M	14	Recurrent allergic rhinitis, recurrent herpes labialis	472	35	73
16	M	14	Recurrent rhinitis, Helicobacter gastritis, hypogammaglobulinemia	488	162	73
17	F	41	Recurrent gastritis, COPD, hypogammaglobulinemia	441	88	450
18	F	60	Polyarthralgia, hypogammaglobulinemia	437	257	234
19	F	23	Recurrent mild respiratory infections (three per year, one with antibiotic therapy)	411	93	187
20	F	69	Lichen ruber of the oral mucosa	227	89	140
21	M	53	Hypogammaglobulinemia, MGUS, DVT lower extremities with pulmonary infarction	435	54	136
22	F	51	COPD	459	38	78
23	F	63	Diabetes II, recurrent gastritis	459	91	41

Normal range (mg/dl)				815–1784	93–287	108–237
**(B)**						
24	M	50	Gastrointestinal protein loss, malabsorption, generalized edema, hypogammaglobulinemia, intestinal villous atrophy 3 pneumonias in the last 3 years, recurrent bacterial bronchitis, vitiligo	<50	53	183
25	M	20	3 pneumonias in the last 3 years, recurrent bacterial bronchitis, vitiligo	<50	<8	<6
26	M	27	Recurrent bacterial bronchitis ≥2 per year, recurrent folliculitis, hypogammaglobulinemia	<50	<8	41
27	F	27	Recurrent sinusitis for 3 years, 4 pneumonias, arthralgias, rhinitis	<50	<7	<7
28	M	59	One pneumonia shortly before diagnosis, recurrent sinusitis for years	<50	11	29
29	F	47	Rec. bronchitis for 10 years, bronchiectasis, pansinusitis for 8 years, 3 pneumonias	56	<7	8
30	F	46	Recurrent pneumonia (≥2 per year), total 12 pneumonias	69.4	243	345
31	M	13	Recurrent pneumonia	76	<8	50
32	M	15	Recurrent pneumonia for 6 years, malabsorption, protein-loosing enteropathy	164	7	<6
33	M	39	Chronic diarrhea, herpes zoster reactivation, gastrointestinal campylobacter jejuni infection, malabsorption	188	<7	<6
34	F	55	Recurrent pneumonia since childhood, recurrent bronchitis, otitis media, sinusitis for 5 years	199	<8	49
35	F	57	Hypogammaglobulinemia, head x-ray abnormalities suspicious of multiple myeloma	204	11	34
36	F	62	Arthritis of the hip, hypogammaglobulinemia, rec. bronchitis, rhinitis for 10 years	206	<7	51
37	F	24	Hypogammaglobulinemia, recurrent diarrhea, one pneumonia with pleuritis, genital condyloma	210	<7	33
38	F	32	Splenectomy, recurrent bacterial bronchitis, sinusitis, hypogammaglobulinemia	217	<8	33
39	M	40	Total of 13 pneumonias, recurrent bronchitis (≥5 per year) since 1978	217	<8	47
40	F	43	Hypogammaglobulinemia, recurrent bronchitis, sinusitis since childhood	219	44	26
41	F	34	Giardia lamblia enteritis, recurrent bronchitis and sinusitis for years, vitiligo	239	<8	47
42	M	29	Pneumococcal meningitis in 1976, hypogammaglobulinemia, recurrent pneumonia, epilepsy	245	10	<7
43	M	50	Recurrent bronchitis, sinusitis for 3 years, first pneumonia 11 months ago, recurrent diarrhea	267	11	<7
44	M	28	Recurrent pneumonia since 1978 (total of six)	327	<7	84
45	F	71	Recurrent bronchitis during the last 6 years, pulmonary obstruction	367	89	67
46	M	13	Recurrent bronchitis and diarrhea since 1995	374	<8	39
47	M	22	Recurrent diarrhea during the last 15 years, intestinal villous atrophie	379	<7	36
48	F	6	Recurrent otitis media, one pneumonia since 1996	396	242	137
49	M	13	Recurrent ITP during the last 5 years, hypogammaglobulinemia	523	12	13

Normal range (mg/dl)				815–1784	93–287	108–237

**Table 2:** **Patients with hypogammaglobulinemia but no susceptibility to infections show normal IgG antibody responses to a variety of antigens**.

Patient no.	Serum IgG antibodies against bacterial, viral and vaccination antigens							
	Tet-IgG	Di-IgG	Hib-IgG	Pn-IgG	Pn-IgM	TBE-IgG	HBs-Ab	HAV-Ab
				
	IU/ml		ug/ml	Reciprocal titer		VIEU/ml	IU/ml	IU/ml
1	1.44 (4.77)	0.45 (3.77)	2.97	42	58	3186	neg (8615)	
2	4.22	1.03	1.59 (>9)	26	33	158 (417)	218 (25658)	37 (7599)
3	0.43 (5)	0.21 (>1)	1.98	<20 (101)	465 (729)	na	na	na
4	2.38	0.23	1.5	210	214	2702	na	na
5	1.52	0.28	25.01	210	314	1740	na	na
6	0.97 (4.26)	<0.01	0.72 (5.86)	64	102	1050	7784	8800
7	2.1 (13.71)	<0.01 (1.04)	1.29	98 (536)	50 (945)	60 (1040)	neg (698)	neg (3832)
8	na	na	1.27 (32.68)	621	180	na	na	na
9	0.89	0.17	>9	26	270	1375	na	na
10	0.02	0.08	1.88	323	<20	na	na	na
11	1.04	0.06	6.31	96	72	403 (10542)	neg (238)	7592
12	0.31 (23.12)	0.22 (7.37)	0.97 (2.17)	235	113	1936	448	1093
13	4.11	0.01	0.34 (>9)	119 (1429)	181 (811)	312	737	>8800
14	1.88	0.3	2.34	592	714	2683	na	na
15	33	2.37	2.45	336	230	>6500	na	na
16	6.2	0.04 (>15)	8.25	363	39	2701	na	na
17	2.29	<0.01	0.77	277	298	1285	neg	>8800
18	3.49	0.17	0.25 (1.76)	30 (242)	343 (673)	1159	13	2355
19	3.46	0.85	2.11	574	825	5289	34876	1501
20	2.08	0.01	0.17	67 (371)	148 (836)	236	356	na
21	1.28 (6.12)	0.03 (0.17)	na	48 (756)	48 (2189)	26	neg	311
22	5.17	0.05	0.11	348	135	360	na	na
23	0.39 (10.18)	0.03	0.39 (>9)	33 (307)	25 (200)	734	na	na

Normal range	>0.4	>0.4	>1	>200	>100	>310	>100	>100

*Values in parentheses represent IgG antibody responses 6–8 weeks following booster immunization; na = value not available*.

### Flow cytometry and examination of humoral immunity

Lymphocyte subpopulations and B cell subsets were analyzed by flow cytometry using standard protocols with commercially available directly conjugated monoclonal antibodies (anti-CD19 PerCP, Becton Dickinson Austria Ges.m.b.H., Schwechat, Austria; anti-IgD FITC, Becton Dickinson Austria Ges.m.b.H., anti-CD27 PE, eBioscience, Vienna, Austria) and a FACSCalibur (Becton Dickinson Austria Ges.m.b.H.). Data analysis was performed using CellQuest software (Becton Dickinson, Austria Ges.m.b.H.). Serum concentrations of immunoglobulins and IgG subclasses were determined by laser nephelometry using reagents purchased from Siemens-Behring Division (Siemens Healthcare Diagnostics GmbH, Vienna, Austria). Serum levels of IgG and IgM antibodies against bacterial and viral antigens were determined using commercially available enzyme-linked immunosorbent assay (ELISA) kits for IgG antibodies against tetanus (VaccZyme Tetanus Toxoid IgG EIA, The Binding Site GmbH, Schwetzingen, Germany) and diphtheria toxoid (VaccZyme Diphtheria Toxoid IgG EIA, The Binding Site GmbH), pertussis (*Bordetella pertussis* IgG ELISA – VIROTECH, Sekisui Virotech GmbH, Rüsselsheim, Germany), TBEV TECHNOZYM^®^ FSME (TBE) lgG (CE), Technoclone GmbH, Vienna, Austria), mumps (Mumps-IgG ELISA Genzyme/Virotech, Sekisui Virotech GmbH), measles (Measles-IgG ELISA – VIROTECH, Sekisui Virotech GmbH), and rubella virus [ETI-RUBEK-G Plus, DiaSorin S.p.A., Saluggia (VC), Italy], VZV (VZV-IgG ELISA – VIROTECH, Sekisui Virotech GmbH), HSV-1 (anti-HSV-1 (gC1)-ELISA (IgG), Euroimmun AG, Lübeck, Germany) and HSV-2 1 (anti-HSV-2 (gG2)-ELISA (IgG), Euroimmun AG), hepatitis B virus (Enzygnost Anti-HBs II, Siemens Healthcare Diagnostics GmbH), hepatitis A virus (Enzygnost Anti-HAV, Siemens Healthcare Diagnostics GmbH), and *Haemophilus influenzae* type b (Hib) (VaccZyme™ Hib-IgG The Binding Site GmbH) or in-house produced ELISAs for IgG and IgM antibodies against 23-valent pneumococcal capsular polysaccharide and 4-valent meningococcal polysaccharide as previously described (10).

### Examination of IgG antibody response to a primary antigen in patients with CVID

The major part of the CVID cohort studied has been vaccinated against TBEV before the observation period, as it is usual practice in Austria, and for this reason TBEV could only be applied as a primary antigen in a subgroup of our patients. Ten patients with CVID were vaccinated against TBEV (FSME-Immun-Inject, Baxter AG, Vienna, Austria, a commercially available licensed vaccine), a primary viral antigen for these patients. The patients were vaccinated twice (4-week interval between the two immunizations), followed by a third booster vaccination 6–12 months following the first immunization. During their participation in this vaccination study, the 10 CVID patients received IVIG lots with a relatively low TBEV-IgG antibody content. Serum IgG antibodies were determined by ELISA as previously described (10) before vaccination, 4–6 weeks after the second as well as before and 4 and 8 weeks after the third vaccination. Healthy controls with a positive TBEV vaccination history and CVID patients receiving IVIG-replacement therapy but no vaccination served as controls.

### Statistical analysis

Statistically significant differences between study groups were calculated using the non-parametric two-tailed Mann–Whitney *U*-test. Results are depicted using box plot diagrams, with the median represented by a cross, the interquartile range (IQR) represented by the box, 5- and 95-percentile values represented by the whiskers, and minimum and maximum values represented by circles.

## Results

### Patients with hypogammaglobulinemia can be differentiated based on their capacity to produce IgG antibodies

The hypogammaglobulinemic patients presented in this study could be divided into two groups according to clinical and immunological characteristics. First of all, most patients with CVID showed a marked susceptibility to bacterial infections [Table 1 (B)] that normalized following initiation of IVIG or SCIG therapy. In contrast, patients with HIAP displayed no or only mild susceptibility to infections, as can be seen from the clinical characterization depicted in Table 1 (A), and did not require immunoglobulin replacement therapy. HIAP patients that were followed for many years did not show a worsening of their clinical condition [follow-up years, median (IQR) 3.8 (0.3–9.8), *n* = 23]. Furthermore, the study population could be differentiated on the basis of their IgG antibody formation capacity, which correlated with the presence of clinical symptoms. In HIAP patients with little or no susceptibility to infections, the intact IgG antibody response was demonstrated by measuring serum IgG antibody levels against a variety of different antigens (bacterial toxins, polysaccharide antigens, viral antigens). In addition, 14 HIAP patients received a booster vaccination against at least one of the vaccination antigens tested and showed a normal IgG antibody response thereafter (Table 2), while other patients in this group showed high IgG antibodies upon first examination because of relatively recent booster vaccinations that were performed according to current immunization recommendations.

**Figure 1:**
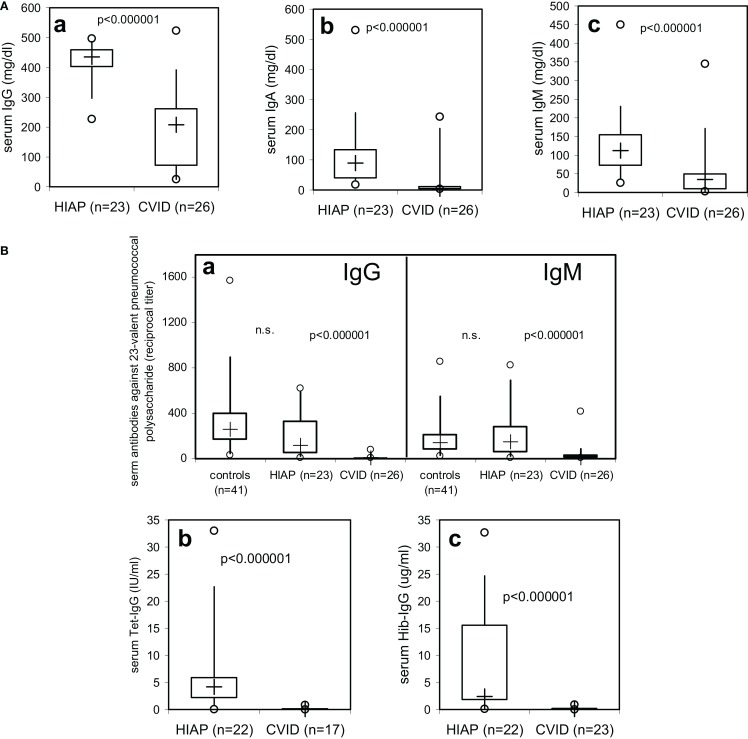
**Serum-immunoglobulin (A) and serum-IgG antibody levels against tetanus toxoid, haemophilus influenzae type b (Hib), and 23-valent pneumococcal polysaccharide (B) in CVID patients and patients with hypogammaglobulinemia but intact antibody production (HIAP)**. Box plot diagrams indicate the median (+), interquartile range (box), percentiles 5 and 95 (whiskers), and minimum and maximum values (circles). Statistical comparison between the two study groups was performed using the Mann–Whitney *U*-test.

Serum-immunoglobulin levels were significantly lower in CVID patients as compared to HIAP patients (Figure 1A), with a substantial overlap of approximately 25% in serum IgG levels between the asymptomatic HIAP group and the CVID patients examined before immunoglobulin treatment (Figure 1A, a). In contrast, median IgG antibody levels against pneumococcal and Hib polysaccharides (without prior vaccination) in CVID patients were more than one log range below the levels observed in HIAP patients without prior vaccination (Figure 1B), with very little overlap between the two groups: in the CVID group, only 5/26 had detectable IgG antibodies against 23-valent pneumococcal polysaccharide (Figure 1B, a) but the median titer of the group was significantly below that observed in healthy controls without vaccination, with only a very small (5%) overlap (Figure 1B, a). Furthermore, 95% of the CVID patients showed IgG serum antibody levels against tetanus toxoid that were below 0.4 IU/ml despite a positive immunization history (Figure 1B, b), while all HIAP patients with a positive tetanus immunization history (21/22 tested) had clearly detectable tetanus-IgG antibodies (≥0.4 IU/ml). All CVID patients tested had Hib-IgG antibodies below 1 μg/ml (Figure 1B, c), antibody levels considered sufficient for long-term protection, and 75% of the patients had no detectable tetanus-IgG or Hib-IgG antibodies at all (Figure 1B). In the CVID group, the IgG antibody deficiency correlated with a markedly increased susceptibility to infections [Table 1 (B)].

### B-memory cell differentiation in CVID and HIAP patients

**Figure 2:**
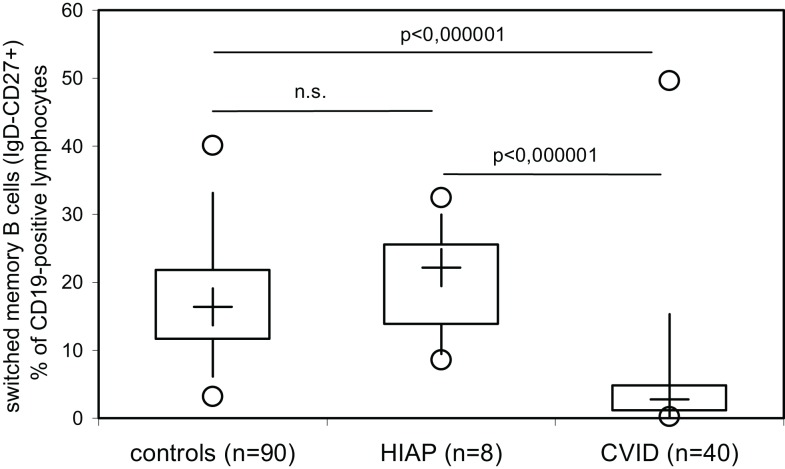
**Switched memory B cells (IgD-negative, CD27−, and CD19-positive peripheral blood lymphocytes) were determined by three-color flow cytometry in healthy adult controls, patients with hypogammaglobulinemia but intact antibody production (HIAP) and CVID patients**. Box plot diagrams indicate the median (+), interquartile range (box), percentiles 5 and 95 (whiskers), and minimum and maximum values (circles). Statistical comparison between the study groups was performed using the Mann–Whitney *U*-test.

Our findings confirm previously published evidence that the majority of CVID patients or CVID patients as a group have decreased switched B-memory cells, as was originally reported by Warnatz et al. more than 10 years ago in order to identify subgroups of CVID patients (11), which was subsequently reevaluated in many publications. Recently, it was reported that low numbers of switched memory B cells correlate with infectious complications in pediatric patients with CVID (12, 13). We thus investigated whether or not patients with hypogammaglobulinemia but intact antibody production can be differentiated from CVID patients on the basis of the number of switched B-memory cells in peripheral blood. While HIAP patients showed levels of switched B-memory cells (CD19^+^CD27^+^IgD^−^) that were comparable to healthy controls examined in parallel, switched B-memory cells were significantly decreased in CVID patients as a group. However, a 25–50% overlap in switched B-memory cells could be found between CVID patients and healthy controls (Figure 2), indicating that a considerable proportion of CVID patients have switched memory B cells within the range of healthy controls, and that low numbers of this B cell subset can also be found in healthy individuals.

### IgG antibody response to a primary antigen is defective in CVID patients

**Figure 3:**
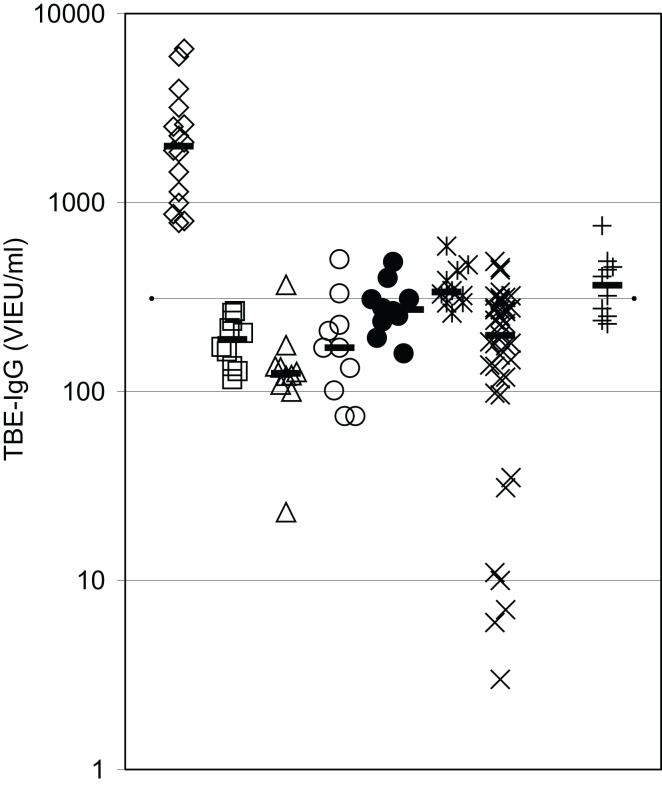
**IgG antibody response to a primary antigen is defective in CVID patients**. Serum IgG antibodies against tick-borne encephalitis virus (TBEV) vaccine were determined by ELISA. Diamonds = healthy controls with a positive TBE vaccination history (*n* = 16); squares = CVID patients before TBEV vaccination (*n* = 10); triangles = CVID patients 4–6 weeks after second vaccination (*n* = 10); open circles = CVID patients before third vaccination (*n* = 10); closed circles = CVID patients 4 weeks after third vaccination (*n* = 10); asterisques = CVID patients 8 weeks after third vaccination (*n* = 10); X = IVIG-treated CVID patients without TBEV vaccination (*n* = 39); crosses = TBEV-IgG antibodies in IVIG lots (diluted to 1000 mg/dl) used during vaccination study (*n* = 10); dotted line = detection limit for positive TBEV-IgG antibodies; median values of the respective groups are indicated by horizontal bars.

IgG antibody response to a primary antigen, TBEV vaccine, was reevaluated in 10 CVID patients receiving IVIG-replacement therapy with lots containing low TBEV-IgG antibody levels. The results depicted in Figure 3 confirm the presence of a substantial defect in IgG antibody production in these patients. Only 1 of the 10 vaccinated patients showed slightly higher TBEV-IgG antibody levels 2 months after the third vaccination as compared to patients receiving IVIG therapy without TBEV vaccination. In all vaccinated CVID patients, TBEV-IgG following the third vaccination was well below the minimum levels observed in healthy adult controls with a positive history of TBEV vaccination (Figure 3).

### Defective IgG antibody production despite normal total serum IgG (case report)

A male patient, aged 39 years, was referred for immunological evaluation because of IgA deficiency, recurrent respiratory tract infections (recurrent otitis media and sinusitis, four infectious episodes that required oral antibiotic therapy during the previous winter including his first pneumonia), lymphadenopathy of the mediastinum and hilus (bronchoscopy revealed a histologic picture compatible with the diagnosis of sarcoidosis). He had received no treatment for his lung problems yet, in particular, never any immunosuppressive therapy. Recent vaccination history included revaccinations against TBEV, dT, and hepatitis A and B. Immunological characterization revealed IgA deficiency associated with IgG2–IgG4 subclass deficiency and low to undetectable IgG antibodies against 14 of 15 antigens tested (Table 3). The only significant IgG antibodies found were against rubella virus as a result of previous infection, presumably during childhood. Total serum IgG levels were normal as were his serum IgM-levels. Upon revaccination with 10 different antigens (including vaccination against poliovirus serotypes I, II, and III), IgG antibody responses against seven of seven antigens tested were missing (Table 3; the seven antigens tested included 23-valent pneumococcal polysaccharide and 4-valent meningococcal polysaccharide). IVIG-replacement therapy was initiated, which led to a complete normalization of his susceptibility to infections during a 3.5-year follow-up. B-memory cells (CD19^+^CD27^+^ switched- and IgM-memory B cells) were absent in peripheral blood; no indication of defective cell-mediated immunity could be found. Other PID disorders were excluded by sequence analysis (Illumina technology performed on a MiSeq bench-top next generation DNA sequencer) of PID genes listed in the 2011 IUIS classification (9).

**Table 3:** **Defective IgG antibody formation against a variety of antigens in a patient with normal serum IgG levels and increased susceptibility to infections**.

Age	Patient			Normal range
	At first examination	After vaccination°	Under IVIG	
	39 years	39 years 5 months	40 years 1 month	
**A. SERUM-IMMUNOGLOBULIN LEVELS (mg/dl)**				
IgG	851	737	1649	(790–1700)
IgA	<6	<6	<6	(76–450)
IgM	100	89	166	(90–350)
IgG1	683	573	1160	(500–880)
IgG2	102	96	400	(150–600)
IgG3	61	51	62	(20–100)
IgG4	<5	<6	<7	(8–120)
**B. SERUM ANTIBODIES AGAINST BACTERIAL AND VIRAL ANTIGENS**				
Tet-IgG (IU/ml)	0.14	0.19°	4.05	>0.4
Di-IgG (IU/ml)	0.08	0.05°	0.73	>0.4
pn23-antibodies (reciprocal titer)
IgG	<20	<20°	525	>200
IgM	<20	42°	75	>100
Hib-IgG (ug/ml)	0.19	0.24°	5.64	>1
Mumps-IgG (VE)	1.6	n.a.	11.2	>11
Measles-IgG (VE)	1.8	n.a.	43.7	>11
Rubella-IgG (IU/ml)	>176.6	n.a.	>182.4	>10
Pertussis-IgG (VE)	2.8	n.a.	13.8	>10
VZV-IgG (VE)	2.6	n.a.	35.9	>11
HSV-1-IgG (VE)	20.7	n.a.	n.a.	>20
HSV-2-IgG (VE)	2.1	n.a.	n.a.	>11
TBEV-IgG (U/ml)	41	27°	444	>310
HBs-Ak (mE/ml)	neg°	neg°	1167	>100
HAV-Ak (IU/L)	neg°	neg°	4049	>100
4-Men-antibodies (reciprocal titer)
IgG	<20	<20°	81	>100
IgM	<20	<20	<20	>50

*Eight to twelve weeks after booster vaccination against hepatitis A and B, Hib, TBEV, Pn23, DiTetPertPolio and first vaccination with tetravalent meningococcal polysaccharide vaccine*.

### Decrease in delay of CVID diagnosis over the last decade

**Figure 4:**
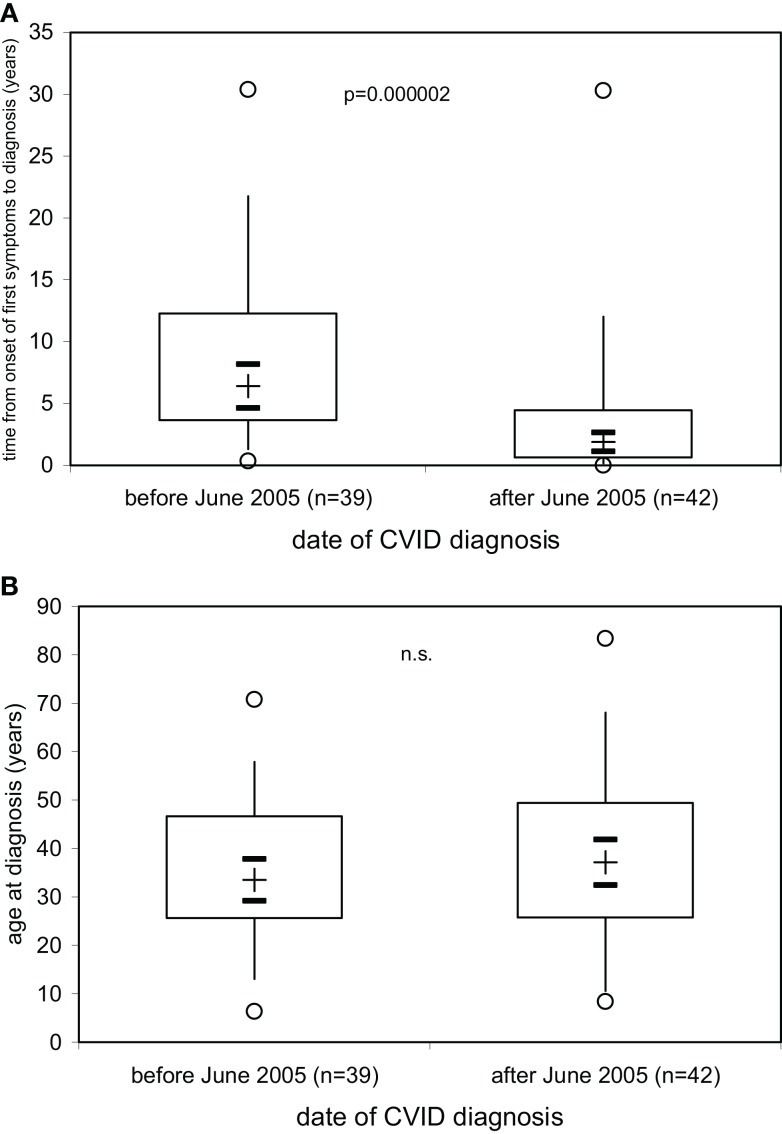
**Decrease in delay of CVID diagnosis over the last decade**. The time (years) between date of onset of first symptoms and date of diagnosis. **(A)** and the age at diagnosis **(B)** is given for 81 patients with CVID divided into two groups according to whether diagnosis was made before (*n* = 39) or after (*n* = 42) June 1st, 2005. Box plot diagrams indicate the median (+), interquartile range (box), percentiles 5 and 95 (whiskers), and minimum and maximum values (circles). Statistical comparison between the two groups was performed using the Mann–Whitney *U*-test. The 95% confidence interval for the median calculated according to McGill et al. (14) is indicated by horizontal bars.

The results presented above indicate that the decision to start immunoglobulin replacement therapy should not be based on the finding of hypogammaglobulinemia but rather on the demonstration of defective IgG antibody formation. A more precise laboratory definition as a rationale to start immunoglobulin replacement therapy is particularly needed in early diagnosed PID patients before a massive history of infectious episodes and/or infectious complications such as bronchiectases have developed. As a result of ongoing campaigns to raise awareness for PID more and more patients with PID are diagnosed with a relatively short or, optimally, no history of clinical symptoms, in particular recurrent infectious episodes. We thus examined whether or not the time between the onset of first symptoms and the date of diagnosis of CVID became shorter in the patients diagnosed at our institution over the last 25 years. On 81 patients diagnosed with CVID according to the criteria established by ESID (4, 5) between June 30th, 1981 and September 15th, 2014, information was available to determine the time (years) between date of onset of first symptoms and date of diagnosis (Figure 4A) as well as the age at diagnosis (Figure 4B). The results presented in Figure 4A show that in our CVID patients diagnosed before June 1st, 2005 (*n* = 42), a median of 6.4 years (IQR, 3.6–12.3) elapsed between onset of first symptoms and diagnosis and initiation of therapy, while during the decade following June 1st, 2005 this diagnostic delay was significantly shorter [median years (IQR]) 1.9 (0.6–4.5), *p* = 0.000002). The age at diagnosis was comparable between the two groups of patients diagnosed before and after 2005 (Figure 4B), showing that age at diagnosis in CVID patients is not a suitable parameter to determine how long clinical symptoms existed before diagnosis.

## Discussion

As a result of ongoing campaigns to raise awareness for PID more and more patients with PID are diagnosed with a relatively short or, optimally, no history of clinical symptoms, in particular recurrent infectious episodes. At our institution, CVID patients diagnosed during the last decade showed a reduction in diagnostic delay by a median of 70% as compared to patients diagnosed earlier. While this development is certainly desirable, it also raises the problem that diagnostic criteria (4, 5) that include mandatory clinical symptoms will not be fulfilled if the patient is treated as early in life as possible. This is especially true in those forms of primary immunodeficieny that lack a definitive diagnosis, e.g., through demonstration of a mutation in the causative gene, as is the case in the majority of patients with CVID. In the absence of clinical symptoms, the currently used diagnostic criteria for CVID mainly rely on the finding of hypogammaglobulinemia. The results presented in this study show that defective IgG antibody response to a variety of antigens rather than hypogammaglobulinemia correlate with the need for immunoglobulin replacement therapy in individual patients to normalize a preexisting susceptibility to infections. As individual patients with severe IgG antibody deficiency might show IgG antibodies against selected antigens with contact, e.g., infection early in life, as many specificities as possible should be examined. IgG antibody responses after infection, natural exposure, or booster immunization and against T-dependent and T-independent, viral and bacterial, protein and polysaccharide antigens, either vaccine-induced or infection-induced, should be tested to provide optimal diagnostic certainty when the option of a definitive diagnosis of CVID does not exist yet. Primary IgG antibody responses should also be a diagnostic option in selected patients with inconclusive evaluation of booster IgG responses or to reevaluate IgG antibody production in patients already receiving immunoglobulin replacement therapy.

In 2014, ESID proposed new diagnostic criteria for CVID that include either defective antibody production (defined as a poor antibody response to vaccination or absent isohemagglutinis) or low switched B-memory cells as a mandatory decisive factor (5). Our findings confirm previously published evidence that the majority of CVID patients and/or CVID patients as a group have decreased switched B-memory cells, as was originally reported by Warnatz et al. more than 10 years ago in order to identify subgroups of CVID patients (11). However, a considerable overlap (between 25 and 50%) in switched B-memory cell distribution could be found between CVID patients and normal healthy controls, suggesting that CVID patients can present with normal numbers of this B cell subset and that low numbers of switched B-memory cells can also be found in individuals with a normal IgG antibody production capacity, thus questioning the usefulness of this parameter as a rationale to start immunoglobulin replacement therapy without testing IgG antibody responses. Further studies are required to answer the question whether very low switched B-memory cells (below the range observed in healthy controls and well below the 70%-of-normal-cut off as proposed in the 2014 ESID registry diagnostic criteria (5) are invariably associated with a clinically relevant defect in IgG antibody production.

To further explore the antibody formation capacity of CVID patients, we examined the IgG antibody response to a primary antigen. Since ongoing immunoglobulin replacement therapy complicates the determination of IgG antibody responses after vaccination, we choose TBEV vaccine, an antigen suitable for primary immunization in individuals not previously vaccinated, as IgG antibody titers are frequently low in IVIG products due to the use of a large share of plasma from the US, where in contrast to middle Europe, TBEV immunization is not frequently employed (15). Previous studies proposed the use of TBEV vaccine to study antibody response to booster vaccination in patients receiving immunoglobulin replacement therapy (16), but primary TBEV antibody response has not been studied in IVIG-treated CVID patients so far. Since it is known that different IVIG lots contain different TBEV antibody contents depending on the country of plasma origin (15), we examined the lots of IVIG that were used in our patients undergoing TBEV vaccination to confirm their low TBEV antibody content. The results obtained extend our previous knowledge of a substantial defect in antibody production in CVID by showing a defective primary IgG antibody response in CVID patients under IVIG therapy. These findings confirm and extend a previous study showing defective booster antibody responses in CVID patients under IVIG treatment on the level of defective antibody forming cells examined by ELISPOT and plasmablasts examined by flow cytometry of peripheral blood B cells (17).

The concept that defective IgG antibody production rather than hypogammaglobulinemia shows the requirement for IgG replacement is supported by findings in an adult patient with IgA deficiency and IgG2–IgG4 deficiency who, despite his normal total IgG levels, displayed a marked defect in the formation of IgG antibodies against both T-dependent and T-independent antigens, associated with normalization of infectious susceptibility under IVIG-replacement therapy. While patients with agammaglobulinemia and complete lack of antibody production are well known to be susceptible to infections, equivalent clinical symptoms can develop in patients with normal levels of total serum IgG but defective IgG antibody production against clinically relevant infectious organisms such as patients with selective polysaccharide antibody deficiency (SPAD). SPAD is a well-recognized primary predominant antibody deficiency (9) that was first described by Umetsu et al. in patients with IgG2-subclass deficiency (18). It was later discovered that even patients with normal IgG subclass levels can fail to produce antipolysaccharide antibodies (19) and that IVIG-replacement therapy effectively prevents infections in these patients, despite their normal levels of total serum IgG (20). The patient described here has IgA, IgG2, and IgG4 deficiency in the presence of normal total serum IgG levels, but in contrast to previously published patients with IgG subclass deficiency and selective anti-polysaccharide deficiency, has defective IgG antibody response against seven of seven tested vaccination antigens, i.e., against T-dependent protein antigens and T-independent polysaccharide antigens, thus closely resembling the severe antibody defect seen in CVID. Comparable to CVID, IVIG-replacement therapy led to a normalization of his susceptibility to respiratory tract infections, and his lung abnormalities (histologically classified as sarcoidosis) remained stable or even improved slightly.

In conclusion, the findings presented suggest that in order to facilitate early treatment before recurrent infections may lead to organ damage the antibody formation capacity should be examined in hypogammaglobulinemic patients and the decision to treat should be based on the demonstration of defective IgG antibody formation against all or the vast majority of multiple different antigens tested. How many different IgG antibody specificities have to be tested has to be determined in subsequent studies. A more precise definition of the laboratory findings providing a rationale to start immunoglobulin replacement in predominantly antibody deficiency is particularly helpful in early diagnosed PID patients, lacking a definitive genetic diagnosis and/or in patients with an atypical clinical presentation.

## Author Contributions

HW and ME were the principal investigators, provided laboratory resources, analyzed clinical and immunological data, wrote the first manuscript draft together, critically participated in all revisions of the manuscript and take primary responsibilities for the paper. VT and JL performed the vaccination study of primary antibody responsiveness, provided clinical patient data, participated in data analysis and interpretation, and critically reviewed the initial draft and all revisions of the manuscript.

## Conflict of Interest Statement

The authors declare that the research was conducted in the absence of any commercial or financial relationships that could be construed as a potential conflict of interest.
